# Experiments on real-life emotions challenge Ekman's model

**DOI:** 10.1038/s41598-023-36201-5

**Published:** 2023-06-12

**Authors:** Sara Coppini, Chiara Lucifora, Carmelo M. Vicario, Aldo Gangemi

**Affiliations:** 1 Institute of Cognitive Sciences and Technologies, National Research Council, Rome, Italy; 2 Department of Cognitive Science, University of Messina, Messina, Italy; 3 Department of Philosophy and Communication, University of Bologna, Bologna, Italy

**Keywords:** Psychology, Human behaviour

## Abstract

Ekman's emotions (1992) are defined as universal basic emotions. Over the years, alternative models have emerged (e.g. Greene and Haidt 2002; Barrett 2017) describing emotions as social and linguistic constructions. The variety of models existing today raises the question of whether the abstraction provided by such models is sufficient as a descriptive/predictive tool for representing real-life emotional situations. Our study presents a social inquiry to test whether traditional models are sufficient to capture the complexity of daily life emotions, reported in a textual context. The intent of the study is to establish the human-subject agreement rate in an annotated corpus based on Ekman's theory (Entity-Level Tweets Emotional Analysis) and the human-subject agreement rate when using Ekman's emotions to annotate sentences that don’t respect the Ekman’s model (The Dictionary of Obscure Sorrows). Furthermore, we investigated how much alexithymia can influence the human ability to detect and categorise emotions. On a total sample of 114 subjects, our results show low within subjects agreement rates for both datasets, particularly for subjects with low levels of alexithymia; low levels of agreement with the original annotations; frequent use of emotions based on Ekman model, particularly negative one, in people with high levels of alexithymia.

## Introduction

Emotions have always been a source of reflection and interest from human beings: they have studied the philosophical, scientific, artistic and literary perspectives on emotions for centuries^1–3^. The numerous theories that emerged within various disciplines attempt to explain the origin, function, and other aspects of emotions, such as the relationship between the behaviour of the subject feeling the emotion, and the surrounding environment. Emotion theories span across categorical emotional models, dimensional emotional models, as well as other, more recent ones.

Categorical emotional models (and appraisal theories) define emotions as discrete categories, defined as ‘primary emotion types’ or ‘basic emotions’, assuming their innate and universal nature, such as happiness, sadness etc. According to the mostly used models, primary emotion types are either five, six or seven^4–6^. Generally speaking, the criteria for the definition of basic emotions are cognitive—the most famous being the OCC model^7–10^—linguistic^11^; or expressive. In the latter case, the primary emotions are defined by clustering physical expression types (in the face, in the gestures, in the body, etc.), in different cultures. This is the case of Paul Ekman’s model^4^. Along a series of studies, Ekman found high agreement across members of diverse Western and Eastern literate cultures on selecting emotional labels that fit facial expressions. Emotions he found to be universal are happiness, sadness, anger, disgust, surprise, and fear.

Dimensional emotional models define emotions according to predefined measures, which typically include valence, arousal, and control. Acknowledged dimensional theories can be distinguished into circumplex models^12,13^, and vector models^14^. While according to the circumplex model the dimensions of arousal and valence are distributed in a circular pattern, in the vector model the emotional direction is determined by an underlying arousal and a binary choice of valence (positive or negative).

Other innovative or more recent theories include new multidisciplinary approaches and new perspectives in emotion research, especially involving affective psychology and neuroscience. An example of the former is the Emotional theory of social psychology^2^, according to which emotions cannot be detached from their moral component: moral emotions are seen as means to strengthen the social cohesion of the group, and for this reason they have an evolutionary development. In the latter case, the debate on the genesis and role of emotions within neuroscience has been animated in recent years by the Constructed Emotion theory, presented by the neuroscientist Lisa Feldman Barrett in her book How Emotions Are Made^3^. Constructed Emotion Theory concentrates on the nature and genesis of emotions, which is in stark opposition to the dominant theories in neuroscience, since it argues that emotions cannot be detected through facial expressions or any other physiological measurement, and it is not possible to state that there are “universal” emotions across people, nations, or cultures. Each one of us constructs its own emotions based on personal experiences, which can eventually be shared with others. Emotions are not “reactions” to external events: the experience of an emotion is a “simulation” or prediction of the appropriate way for the body to react to an event. The experience of an emotion is a prediction of the brain of what it thinks it might happen next. Therefore, emotions are concepts that are constructed by the brain out of pieces of sensory data, knowledge and history of social interactions.

Besides specific research on emotions, there is a large body of emotional knowledge from literature and other art forms.

For example, “unnatural emotions” are defined either as (1) narratives that have a defamiliarizing effect because they are experimental, extreme, transgressive, unconventional, nonconformist, or out of the ordinary, or as (2) physically impossible scenarios and events^15,16^. For instance, a “logically impossible” emotion, which goes against principles ruling the real world, could be the atypical feeling of offence and rage by the death of a young classmate in Julian Barnes’s The Sense of an Ending: “Now he had offended us by making a name for himself with an early death”^17^.

For its originality and uniqueness in the contemporary literary panorama, The Dictionary of Obscure Sorrows (DOS) by John Koenig, represents a “compendium of new words for emotions. Its mission is to shine a light on the fundamental strangeness of being a human being—all the aches, demons, vibes, joys and urges that are humming in the background of everyday life”^18^. Beyond what has been given a name and classified in different ways, there is more that it is not classified yet, but which is worth writing to celebrate the intrinsic complexity, which characterises human existence, and makes it extraordinary. The text itself is structured like a dictionary, so that new words invented by the author are associated with definitions that concern our inner life. Due to their innovative and extraordinary nature (understood as out of the ordinary), these emotions could be called “undefined”, with respect to common sense vocabulary and traditional models. They might happen to be “unfamiliar emotions”, but we might have already experienced some of them, without realising that they can be hardly classified by traditional emotion types. It is a matter of relevance for emotion research to access the complexity and detail provided by emotion descriptions that are not supported by traditional models.

Beyond the specific models aimed at defining emotions, recent studies denote an important correlation between the ability to identify, understand and describe emotions, and the affective and engaging power they have over ourselves^19,20^. In other words, affect labelling can help people feel better by dampening negative emotions, while also heightening positive emotions, but only if there a subject has a good ability to put feelings into words, after having identified their emotional experiences. To do that, for what concerns negative emotions, they must self-reflect not only on what their feelings are, but also on what may be causing their emotions, which eventually leads to feeling better. As for positive emotions, the researchers hypothesise that just by stating your feeling, you can easily focus your attention on it, identify what specific type of emotion is, and eventually self-reflect on it, making the feeling last longer. This leads to another relevant aspect that has been pointed out: affect labelling can be more effective (for emotion management and awareness) when using specific words like ‘amused’ or joyous, instead of using generic words such as ‘happy’. Further, research has shown that labelling positive emotion with accurate terms helps in coping better with negative emotional states, such as stress, for the reasons mentioned^21^. On the other hand, people having difficulty identifying and labelling their emotional experiences may be inclined to experience negative emotions more frequently and with more intensity^22,23^.

In this line, along with existent literature on the matter [e.g., ^24^],our research focus is whether emergent or undefined emotions can be described with traditional models. For this reason, we have used both a corpus (Entity-Level Tweets Emotional Analysis) annotated with Ekman's theory emotions and a corpus (The Dictionary of Obscure Sorrows) annotated with newly created emotions, to establish the human subject agreement rate when annotating sentences using classical emotional labels.

The two datasets express emotions linked to specific circumstances of everyday life which can be associated with complex/circumstantial/undefined emotions.

However, the first dataset is annotated according to Ekman while the second is expected to contain undefined emotions (hence the newly created labels). This allows us to investigate if the undefined emotions can be reduced to traditional Ekman’s emotions, or if, on the contrary, these emotions are irreducible to the first known ones.

Among the various well-known emotional models that define "basic" emotions understood as universal and possibly innate, the six emotions defined as "basic" by Ekman^1,4^ were selected for this investigation.

We intend to challenge that all emotional experiences can be reduced to the six universal and basic emotions.

In order to test the hypothesis, we have administered a standard questionnaire to a community of people with the same social and cultural background, using a cognitive method, giving particular importance to alexithymia, described as a characteristic or personality trait characterised by a processing disorder affective-emotional that causes difficulties in identifying and describing feelings and emotions^25–28^.

The term alexithymia was introduced by Sifneos in 1973^29^ and can be literally defined as "without words for emotions". It is a personality trait that involves the emotional sphere of the subject, and it is related to a large number of psychological disorders (e.g., depression^30^ and schizophrenia^31^) as well as physical diseases (e.g., psychosomatic illness^32^). The alexithymic subjects present both cognitive (e.g., difficulty in recognizing, describing and distinguishing emotions) and affective (e.g., emotionalizing) deficits^33,34^.

Hence, our research questions can be formulated as follows:

- Are existing emotional models sufficient to describe the complex/circumstantial emotional situations that characterise human experience?
- Can different levels of alexithymia influence the understanding, identification and perception of complex and circumstantial emotions?

While real-life emotional situations reflect a dynamic process that can never be completely reduced to a finite set of categories, yet quantifying the experiential states and the elements that compose them is necessary for research aimed at a rigorous analysis of cognition for designing computational systems.

Our work originally stems from artificial intelligence research, where extensive annotated corpora, formal models, and machine learning are used to detect or predict emotions from text, facial expressions, gestures, etc. Current AI research typically focuses on datasets annotated via the six basic emotions [e.g., ^35,36^] that are universally shared among humans (like happiness, sadness, disgust, fear, anger and surprise). However, inspired by the Dictionary of Obscure Sorrows (DOS) we wonder if Ekman's emotions may not be enough to explain all the emotions we experience in real life situations. In this sense, in the context of an international project (The SPICE project deals with data-driven cultural engagement for social inclusion and empathy development. Its knowledge graph infrastructure includes multiple emotion theories that have been represented in the SPICE Ontology Network to support the automated analysis and sharing of citizen interpretation about works of art), we are enhancing emotion-oriented datasets and lexical resources, in order to investigate everyday emotions with computational means. How can emotional situations be detected, interpreted and categorised, e.g., in text, conversation, or multimodal interaction? We are representing emotion-oriented resources as knowledge graphs, a common format to create interoperable datasets based on formal semantics. An example of interoperable knowledge graphs from either factual or linguistic resources is Framester^37^, which uses a formal cognitive frame semantics^38^.The results from the experiment we present here spot some limits of existing emotion models, and the need to integrate or extend them to represent realistic emotion situations. Starting from the results of this work, an integrated, flexible computational model for emotion situations is under construction.

## Materials and methods

### Participants

Our sample is made up of 114 adults’ participants (over 18 years old), divided in 20 males and 94 females. In our study, we considered a specific age range over 42 years old (gen X), and under 41 years old (gen Z)^39–41^ to have a representative sample of the adult population.

So far, in our sample 53.5% of subjects are 42 years old or more, and 46.5% 41 or less years old. Informed consent form was obtained from all participants trough the online module. The study was conducted according to the guidelines of the Declaration of Helsinki. All experimental protocols were approved by the Ethics Committee of the Department of Cognitive Science, University of Messina (protocol code COSPECS_04_2023).

### Instruments

The first section of our questionnaire consists of the Italian translation of the Perth Alexithymia Questionnaire (PAQ), designed by Black Swan Psychological Assessments Pty Ltd^42,43^ Alexithymia is a multidimensional construct defined on three levels, that are: emotion identification, emotion description and emotion focus. Indeed, it is “comprised of three components: difficulty identifying one’s own feelings (DIF); difficulty describing feelings (DDF); and an externally oriented thinking style (EOT) whereby one tends to not focus their attention on their emotions”^43^.

The PAQ test is made up by 24 items related to 10 subscales, that are Negative-Difficulty identifying feelings (N-DIF); Positive-Difficulty identifying feelings (P-DIF); Negative-Difficulty describing feelings (N-DDF); Positive-Difficulty describing feelings (P-DDF); General-Externally oriented thinking (G-EOT); General-Difficulty identifying feelings (G-DIF); General-Difficulty describing feelings (G-DDF); Negative-Difficulty appraising feelings (N-DAF); Positive-Difficulty appraising feelings (P-DAF); General-Difficulty appraising feelings (G-DAF). The score ranges in a Likert Scale from 1 (strongly disagree) to 7 (strongly agree).

Due to the lack of an Italian translation of the PAQ, following a previous study by Becerra et al.^44^, the English PAQ items have been independently translated into Italian by all the authors of this paper, followed an agreement procedure.

In accordance with the recent literature^45^ which highlights an important difficulty in verbalising emotions in people with alexithymia, the PAQ was included to verify how well the different levels of alexithymia, in healthy subjects, can influence the personal perception and conceptualization of emotions in daily life.

The second section of the questionnaire consists of 20 sentences describing emotional experiences, to be annotated by participants, using either (i) one of the six emotions from the Ekman’s model proposed; (ii) the option “other” which allows either for the verbal specification of an emotion not included in the six proposed, or the specification of more than one emotion among those proposed or other emotions; (iii) the option “no emotion” if they felt like there was no emotion expressed or involved in the emotional experience described in the sentence.

Of the 20 sentences, 10 were taken from the literary resource The Dictionary of Obscure Sorrows (DOS)^18^, and 10 from the annotated dataset ELTEA17^46^, which contains tweets annotated according to Ekman's emotion theory.

ELTEA17 was chosen because its content is close to the entries of The Dictionary of Obscure Sorrows: emotions are not simply described or named, but an emotional context is provided, typically as a situation involving the subject experiencing the emotion; while DOS is a project that aims to create new terms to describe complex emotions and experiences that often don't have a specific name. For example, the term "onism" is used to indicate the sensation of feeling very small compared to the surrounding world, or "sonder" to refer to the awareness that everyone has their own story (Supplementary Table S1 shows the DOS entries that we used in our questionnaire).

Among the annotated tweets of ELTEA17 we have chosen those that are as similar as possible to the structure and type of content of the entries of DOS. Specifically, the tweets were chosen according to the following features: (i) they express a specific situation; (ii) they express a temporal determination (e.g., starting with "when you feel that…”); (iii) they feature some complexity and ambiguity, either in the narration of the subjective emotional experience or in the physical or temporal determination; (iv) they contain generic content, i.e., not related to specific people, places or personal experiences (e.g., “at my brother’s birthday, my mum made me feel sad” was excluded).

Furthermore, both in choosing the tweets and in choosing the DOS entries we tried to include different types of emotions, that is, texts with either a cheerful or sad tone, i.e., situations that cause either pleasant or unpleasant emotions. We have translated the original in the Italian language (Supplementary Table S2).The purpose of this test case is twofold: on the one hand, we intend to verify whether it is possible to “reduce” the situational and complex emotional experiences described by a literary resource, or whether a new model is actually needed to describe them; on the other hand, the purpose is to verify whether the annotation through an existing and well-established model reaches an agreement between the annotators—whether or not it conforms to the annotation originally proposed by ELTEA17—or if it is also insufficient.

### Procedure

The questionnaires were created through the “Google Forms” platform, and administered remotely to the participants, through the main social media channels and social networks, (Facebook, WhatsApp, Instagram etc.). We conducted an online study both to access a large population of users and to avoid a possible influence given by the interaction with the interlocutor. In the construction of our questionnaires, we highlighted various factors, such as: (i) closed questions, that are clearer in the coding; (ii) the time needed to complete (about 10 min); (iii) the syntactic structure; (iv) the semantic ambiguity. Last, our questionnaires were administered in the Italian language. Our questionnaire is composed of 4 pages, in the first page we gathered consent to participate in the study, and demographic variables such as age and gender. Next, the participants were asked to complete the PAQ test (page 2) and provide responses to the proposed emotional sentences related to ELTEA (page 3) and DOS (page 4). We have excluded the possibility of submitting double answers (by deactivating multiple submission) or incomplete answers (making each question mandatory). All participants completed the test correctly.

## Data analysis

We divided our sample in three categories, based on the score obtained in overall alexithymia test^42,43^, that are: (i) Group 1: Scores 1SD or more below the mean are considered “low level of alexithymia”, N = 19; (ii) Group 2: Scores less than 1SD from the mean are considered “average level of alexithymia” N = 81; (iii) Group 3: Scores 1SD or more above the mean are considered “high level of alexithymia” N = 14.

Our results, obtained through the one-way ANOVA test related to the degree of agreement between groups, show that there are not significant differences [ELTEA F(2,26) = 0.649, p = 0.508, η^2^ = 0.051; DOS F(2,25) = 0.715;p = 0.499; η^2^ = 0.054]. However, the degree of agreement within subjects shows important differences, specific to each group.

Distribution of Ekman’s emotions in Alexithymic and no-Alexithymic subjects:

Related to the first category (low level of alexithymia), in the annotation of the entries of the ELTEA dataset there is an agreement with the original annotation only in two cases out of ten, and with an agreement percentage higher than 50% only in one case (ELTEA3, for the emotion "Happiness"). In the DOS dataset, only in one case there is an agreement in the annotation of the participants greater than 50% (DOS6 for the emotion "Happiness"). In the other emotional experiences, the agreement is generally between 21.1 and 47.40%.

About the second group (medium level of alexithymia), on the ELTEA dataset, the results show a greater agreement with the original annotation. In fact, only in 4 out of 10 cases there is no agreement. However, in the cases where agreement does occur, it is between 14.80% and 39.50% in 5 out of 6 cases. In fact, the maximum agreement was reached only in the case of the emotion "Happiness" for ELTEA3 (as in the first group) with an agreement degree of 77.80%. Also for the DOS dataset, the second group shows a good agreement in the annotation. For 7 out of 10 emotional experiences the agreement is around 50%, while in the other cases the agreement is between 17.30 and 37%.

Finally, the third category of participants (high level of alexithymia), the results show an agreement with the annotation of ELTEA in 6 cases out of 10, and an agreement with the annotation of the emotions of DOS equal or greater than 50% in half of the cases. For the annotation of ELTEA, the degree of agreement varies considerably, from a minimum of 28.60% to a maximum of 92.90% (ELTEA3 recorded the highest degree of agreement, this data is confirmed also in the other two groups).

Particularly, for all three groups, there is a lack of agreement with the original annotation of ELTEA dataset for entries ELTEA5, ELTEA6 and ELTEA10.

**Figure 1:**
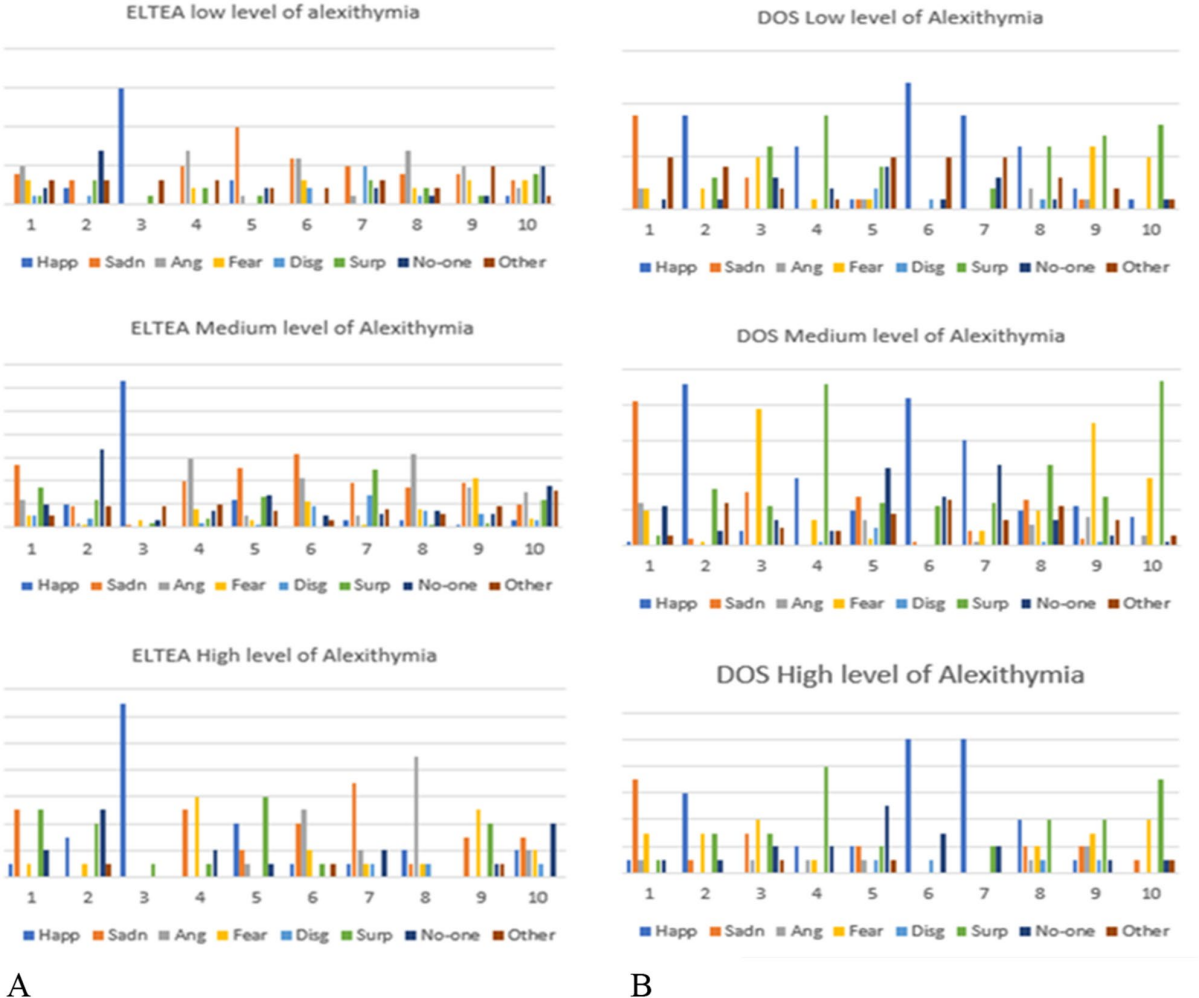
These charts show the distribution of annotated emotion in the entries from ELTEA (**A**) and DOS (**B**), divided for low, medium, and high levels of alexithymia.

Figure 1 shows the distribution of emotions for entries of ELTEA (A) and DOS (B), for each group.

Alexithymia and Ekman’s emotions:

Regarding alexithymia, our general results show a good internal consistency in the reliability of the whole sample: total scale score, overall alexithymia mean: 80.38; SD: 27.52: and 0.94 for Cronbach’s Alpha. For our analysis we used the Kruskal–Wallis test for multiple comparisons, based on the normality test that suggests the use of non-parametric analysis tools (Table 1).

**Table 1:** Shapiro–Wilk test documents no normal distributions for the considered emotions. (*indicates significant differences p ≤ 0.050).

	Sadness	Happiness	Fear	Anger	Disgust	Surprise
Shapiro–Wilk	W = 0.956 p = 0.001*	W = 0.956 p = 0.004*	W = 0.882 p = < 0.001*	W = 0932 p = < 0.001*	W = 0.746 p = < 0.001*	W = 0.949 p = < 0.001*

We compared the frequency of use of emotions and the type of emotions to classify the scenarios proposed in the 3 groups (low, medium and high alexithymia). To control for family-wise error rates post-hoc comparisons were corrected with Bonferroni test. Accordingly, the p-level was considered significant if < 0.016. Overall, we detected a significant difference between our groups (H = 11.99, p = 0.002). The post-hoc comparison documents a higher frequency (p = 0.003) in the use of affective description in individuals with high alexithymia scores (M = 77.78) compared to individuals with low alexithymia scores (M = 40.21). No difference is reported between individuals with low and medium (M = 58.04) alexithymia scores (p = 0.102), as well as between individuals with medium and high alexithymia scores (p = 0.117) (Table 2).

**Table 2:** This table shows that the score of the sum of the answers given is higher in group 3 than in group 1. This suggests that alexithymic subjects generally tend to propose an emotional response more frequently than in the other 2 groups. No difference between 3 and 2 and between 2 and 1. (*Indicates significant differences Bonferroni correction p < 0.016).

Total score	High alexithymia	Medium alexithymia	Low alexithymia
High alexithymia		0.102712	0.003746*
Medium alexithymia	0.102712		0.117336
Low alexithymia	0.003746*	0.117336	

We also found a significant difference in the use of the “other” category, i.e. not classifiable in terms of traditionally-defined affect (H = 11.99, p = 0.002). The post-hoc comparison documents higher (p = 0.003) use of the “other” category in individuals with low alexithymia scores (M = 74.68) compared to individuals with high alexithymia scores (M = 37.21). On the other hand, no difference is reported between individuals with low vs. medium (M = 56.97) alexithymia scores (p = 0.106), as well as between medium and high alexithymia scores (p = 0.116) (Table 3).

**Table 3:** This table shows that the score of the sum of the "other" responses (no affective labels)—is higher in group 1 than in group 3. This result suggests that alexithymic subjects tend to use non-emotional categories to interpret a given scenario less frequently than individuals borderline and non-alexithymic. No difference between 3 and 2 as well as between 2 and 1. (*Indicates significant differences Bonferroni correction p < 0.016).

No-affective labels	High alexithymia	Medium alexithymia	Low alexithymia
High alexithymia		0.106701	0.003866*
Medium alexithymia	0.106701		0.116603
Low alexithymia	0.003866*	0.116603	

This result suggests that individuals with alexithymia tend to use traditional emotional categories to interpret a given scenario much more frequently than non-alexithymic individuals.

A further analysis was performed to compare the frequency in the use of canonical emotions to classify the proposed scenarios in the 3 groups of participants, according to the score provided for all 10 subscales of the PAQ test. Our results show:

(i) A significant difference for the difficulty identifying negative feelings (N-DIF subscale 1) with regard to the emotion of fear (H = 8.832, p = 0.012). Individuals with higher scores on the N-DIF subscale tend to rate scenarios as fearful more frequently (M = 67.03) than non-alexithymic (M = 32.00) individuals (p = 0.011). A no significant trend (0.070) is also reported when comparing individuals with low alexithymia scores with individuals with middle alexithymia (M = 57.21) scores. No significant results were found between individuals with middle and high alexithymia scores (p = 0.522) (Table 4).
(ii) A significant difference for the difficulty describing negative feelings (N-DDF subscale 3) with regard to the overall use of the canonical affective category to describe the proposed scenarios (H = 10.41, p = 0.005). Post-hoc comparison documents a significant difference (p = 0.007) between individuals with high alexithymia scores (M = 72.94) compared to individuals with low alexithymia scores (M = 40.71). No difference was found between individuals with low and medium (M = 57.83) alexithymia scores (p = 0.130), as well as between individuals with medium and high alexithymia scores (p = 0.224) (Table 5). Moreover, for the same subscale, we found a higher frequency (p = 0.009) of individuals with low alexithymia scores (M = 73.84) in describing scenarios as non-canonically affective (other) compared to individuals with high alexithymia scores (M = 42.13). No difference is reported between individuals with low and medium (M = 57.25) alexithymia scores (p = 0.151), as well as between medium and high alexithymia scores (p = 0.223) (Table 6).
(iii) A difference for the General-External oriented thinking (G-EOT subscale 5) with regard to the tendency to the overall use of the canonical affective category to describe the proposed scenarios (H = 12.03, p = 0.002). Post-hoc comparison documents a difference (p = 0.021) between individuals with high alexithymia scores (M = 78.81) compared to individuals with low alexithymia scores (M = 43.88). Moreover, we found a difference between individuals with low and medium (M = 61.81) alexithymia scores (p = 0.025). No difference was found between individuals with medium and high alexithymia scores (p = 0.503) (Table 7). Moreover, for the same subscale, we found a higher frequency (p = 0.020) of individuals with low alexithymia scores (M = 71.186) in describing scenarios as non-canonically affective (other) compared to individuals with high alexithymia scores (M = 36.18). A difference (p = 0.024) is also reported between individuals with low and medium (M = 53.15) alexithymia score. No difference was found between medium and high alexithymia scores (p = 0.506) (Table 8).
(iv) A difference for General-Difficulty describing feelings (G-DDF subscale 7) (H = 6.86 p = 0.03) and General-Difficulty appraising feelings (G-DAF subscale 8) (H = 8.62 p = 0.01) in the use of non-canonical affective terminology (i.e. “other”) less frequently than non-alexithymic subjects. In the subscale 7, people with higher scores of alexithymia (M = 45.14) use “other emotions” less frequently then people with low levels of alexithymia (M = 71.05) (p = 0.043) (Table 9). This result is also confirmed in the subscale 8 (p = 0.022) between people with high (M = 42.50) and low (M = 74.15) levels of alexithymia. No difference is reported between individuals with low and medium (M = 57.70) alexithymia scores (p = 0.371), as well as between medium and high alexithymia scores (p = 0.370) in the subscale 7. No difference is, also, reported between individuals with low and medium (M = 58.53) alexithymia scores (p = 0.340), as well as between medium and high alexithymia scores (p = 0.156) in the subscale 8 (Table 10).
(v) A significant difference (p = 0.01) is related to the use of fear emotion in relation to higher levels of alexithymia in N-DIF (Negative-Difficulty identifying feelings) and G-DIF (General-Difficulty identifying feelings) subscales, using Pearson's Correlation. Furthermore, the generic use of affective terminology increases as the scores on the specific subscales increase. N-DDF (Difficulty describing negative feelings) p = 0.001; G-EOT (General-External oriented thinking) p =  < 0.001; G-DIF (General-Difficulty identifying feelings) p = 0.01; G-DDF (General-Difficulty describing feelings) p = 0.007; G-DAF (General-Difficulty appraising feelings) p = 0.003. In this case, our results show that as the scores on the subscales relevant to the description and evaluation of negative feelings and more generally the description, identification and evaluation of feelings increase, the use of canonical emotional labels increases.
(vi) Chi-square analysis shows a significant difference in ELTEA dataset (Χ^2^ = 10.99; p = 0.004) in the preference for the fear emotion in question number 4, which indicates a high annotation rate of this emotion in group 3 (alexithymics). Regarding the DOS dataset, the Chi-square analysis shows a significant difference (Χ^2^ = 44.98; p =  < 0.001) in question number 5, which indicates a low propensity for category 3 (high alexithymics) to annotate “other emotions” compared to groups 1(low alexithymia) and 2 (medium alexithymia).

**Table 4:** This table shows that there is a greater frequency of the emotion “fear” in alexithymic subjects compared to the control groups. Individuals with high scores on the Difficulty Identifying Negative Feelings (N-DIF) subscale tend to classify scenarios as fearful more frequently than non-alexithymic subjects. (*Indicates significant differences Bonferroni correction p < 0.016).

N-DIF	High alexithymia	Medium alexithymia	Low alexithymia
High alexithymia		0.070373	0.011544*
Medium alexithymia	0.070373		0.522603
Low alexithymia	0.011544*	0.522603

**Table 5:** This table shows that subjects with high scores in the subscale that measures Difficulty describing negative feelings (N-DDF) tend to use affective terminologies for the scenarios proposed more frequently than non-alexithymics. No difference between 3 and 2 as well as between 2 and 1. (*Indicates significant differences Bonferroni correction p = < 0.016).

N-DDF	High alexithymia	Medium alexithymia	Low alexithymia
1 High alexithymia		0.130164	0.007939*
1 High alexithymia	0.130164		0.224005
1 High alexithymia	0.007939*	0.224005

**Table 6:** This table shows that subjects with high scores on the Difficulty Describing Negative Feelings (N-DDF) subscale tend to use non-affective terminology for the scenarios proposed less frequently than for non-alexithymic ones. No difference between 3 and 2 and between 2 and 1. (*Indicates significant differences Bonferroni correction p = < 0.016).

N-DDF	High alexithymia	Medium alexithymia	Low alexithymia
High alexithymia		0.151282	0.009319*
Medium alexithymia	0.151282		0.223248
Low alexithymia	0.009319*	0.223248

**Table 7:** This table shows that participants with intermediate and high scores on the General Externally Oriented Thinking subscale tend to use affective terminologies more frequently than controls. Based on Bonferroni correction, differences in G-EOT subscale are not significant (p < 0.016).

G-EOT	High alexithymia	Medium alexithymia	Low alexithymia
High alexithymia		0.025945	0.021025
Medium alexithymia	0.025945		0.503385
Low alexithymia	0.021025	0.503385

**Table 8:** This table shows that participants with low scores on the General Externally Oriented Thinking subscale tend to describe scenarios as non-canonically affective (other) more than individuals with high alexithymia scores. Based on Bonferroni correction, differences in G-EOT are not significant (p < 0.016).

G-EOT	High alexithymia	Medium alexithymia	Low alexithymia
High alexithymia		0.024778	0.020679
Medium alexithymia	0.024778		0.506031
Low alexithymia	0.020679	0.506031

**Table 9:** This table shows that participants with high scores on the General Difficulty Describing Feelings (G-DDF) subscale tend to use nonaffective terminology less frequently than controls and subjects with intermediate scores. Based on Bonferroni correction, differences in G-DDF are not significant (p < 0.016).

G-DDF	High alexithymia	Medium alexithymia	Low alexithymia
High alexithymia		0.371617	0.043976
Medium alexithymia	0.371617		0.370955
Low alexithymia	0.043976	0.370955

**Table 10:** This table shows that participants with high scores on the General Difficulty Assessing Feelings (G-DAF) subscale tend to use affective terminology more frequently than controls, and subjects with intermediate scores. Based on Bonferroni correction, differences in G-DAF are not significant (p < 0.016).

G-DAF	High alexithymia	Medium alexithymia	Low alexithymia
High alexithymia		0.361273	0.022427
Medium alexithymia	0.361273		0.150606
Low alexithymia	0.043976	0.370955	

## Discussion

This work falls within the research area aimed at investigating the models and formal expressions of emotion description to test their usefulness and efficacy, particularly in relation to people's ability to identify and express emotions themselves.

Starting from the study conducted by Paul Ekman^4^ on the physical expressions (face, body, etc.) of human emotional experience, that led him to the conclusion that happiness, sadness, anger, fear, surprise and disgust are innate human emotions, our study aims to test the abstraction of Ekman's categorical model through affective labelling.

As described within the scientific literature e.g. Ref.^19^, affective labelling is the act of describing emotions verbally, either orally or in written form. Talking about our feelings, or using emotional language to describe what upsets us, has mostly been studied for its effects on emotion regulation, as in attenuating our emotional experiences^19–21^. This last aspect goes beyond the research scope of this work, but could constitute a further development of the study.

To study affective labelling, we used some sentences describing emotional experiences defined as complex or circumstantial, taken from a literary resource (The Dictionary of Obscure Sorrows, DOS)^18^ and the annotated dataset Entity-Level Tweets Emotional Analysis dataset (ELTEA17)^46^. Complex or circumstantial emotions are emotional experiences closely related to the context in which they occur, and they are also referred to as “undefined”, with respect to common sense vocabulary and traditional models. In fact, the proposed texts describe a state of affairs or an inner state of subjects, who find themselves in a specific situation, in which they could live an emotional experience outside of "traditional emotions" (i.e. those defined by consolidated cognitive and linguistic models), which we call an experience of "undefined emotion." They may be "unfamiliar emotions," but we may have already experienced some of them, not realising that they can hardly be classified by traditional emotion types.

On the basis of the recent literature^47,48^ we have also given importance to the personal characteristic of alexithymia in the ability of describing emotions, using the PAQ test in order to distinguish low, medium and high levels of alexithymia.

The basic research questions of this study include:

(i) are existing emotional models and their abstraction level sufficient to describe the complex emotional situations that characterise human experience?
(ii) can different levels of alexithymia influence the understanding, identification and perception of complex and circumstantial emotions with respect to the ability to express and understand one's emotions?

Based on those questions, our research hypothesis aims to understand whether (i) emergent or undefined emotions (i.e., complex and circumstantial emotional situations) can be described with traditional models; and (ii) alexithymia can influence the human ability to detect and categorise emotions. These points will be discussed below.

Traditional model of emotions are inadequate in representing the complexity of emotional experiences.

Overall, results from emotion annotation or affective labelling show low rates of agreement among subjects both for the ELTEA17 dataset and the DOS dataset, especially for subjects with low levels of alexithymia (2 cases of agreement out of 10 for ELTEA17 and only 1case of an agreement percentage higher than 50% for DOS dataset). Furthermore, the cases of major agreement rates between participants (in the case of the DOS dataset) or with the original annotation (in the case of the ELTEA17 dataset) are always the same for each group of participants, i.e. the highest rate of agreement always concerns ELTEA3 and DOS6 for the respective datasets. On the other hand, the low agreement rates vary considerably from group to group, showing that the agreement is quite diverse and generalised, and therefore for the case of the ELTEA dataset it is not necessarily attributable to the original annotation. In other words, if the low agreement rates were concentrated in the same cases for all groups, this could lead us to think that the original notation was simply wrong in those single limited cases; and yet it is not so. Thus if, on the one hand, the low rate of agreement in the affective labelling of the ELTEA17 dataset with its original annotation could lead us to simply hypothesise a lack or superficiality in the original annotation; on the other hand, the low rate of agreement among the participants in the affective labelling also of the DOS dataset and the between-group variation in cases of poor agreement leads us instead to hypothesise a lack, on the part of the traditional models, in representing complex and circumstantial emotional experiences.

Previous studies^24,35,36^ focused on using AI models for emotion recognition, for example developing datasets of emotional facial expressions related to the six basic emotions of Ekman. Although the potential of AI systems in emotion recognition is evident, it seems necessary to develop more advanced AI models capable of capturing the complexity of human emotions. As discussed by Lewinski^49^ these emotion models lack an extended representation of complex emotional experiences. Following Lewinski, in this article we demonstrated that Ekman's emotion model is too rigid to explain the wide range of emotions we experience in everyday life situations, implying the need to improve emotion model and datasets to make computational sense of everyday emotions.

How Alexithymia limits emotional expression to basic emotions:

Results from emotion annotation show that people with higher levels of alexithymia tend to annotate with traditional emotion labels rather than more specific, novel emotions, or a set of emotions. This finding is in line with recent literature showing that alexithymic subjects have difficulty in recognizing emotional expressions on faces^50,51^ and have impaired emotional linguistic processing^33^. In particular, subjects with high levels of alexithymia show difficulties in recognizing specific emotional contexts related to emotion words^52^ and tend to use a less complex vocabulary in emotional narratives referring to themselves and others^53–55^.

The lower levels of agreement among the subjects belonging to the medium alexithymia and low alexithymia samples show a possible tendency of the subjects with greater capacities for identification and emotional expression to look for new ways and new terms to describe emotional experiences. In line with scientific literature e.g. Ref.^19^, affective labelling (i.e., describing emotions verbally) and its effectiveness (in understanding and awareness of emotions) are closely linked to the specificity of the words with which it is noted.

These results allow us to hypothesise that a greater ability to express and understand one’s emotions corresponds to a greater need for specificity in affective labelling, therefore a greater need to search for complex and expressive models to represent the personal emotional panorama. As the results of this study show, traditional or canonical models of emotion description may be insufficient or limiting for people with low alexithymia, who show greater granularity in affect labelling.

As described in literature^19–21^, the ability to express one’s emotions with greater granularity and specificity not only allows us to understand them better, but also to live them positively and be able to regulate one’s experience and emotional burden. More effective emotional regulation and psychological resilience (the ability to bounce back from negative events by using positive emotions to cope) in turn lead to a healthy development of the emotional apparatus and to greater emotional expressiveness, making the possibility of having models of representation of emotions that are specific and expressive. Here lies the relevance of this study also for future developments regarding the need for new emotional models for concrete human personal experience and cognitive development. A hypothetical correlation could be investigated between more expressive emotional models, and the ability to live and experience complex emotional situations, as well as the subjects’ ability to regulate emotions and cope with them.

Secondly, the most interesting result of our study is related to the correlation between alexithymia and emotions.

Our results confirm previous studies^47^ on the ability of alexithymic subjects to recognize and annotate the well-known Ekman Emotions, as well as their low ability in evaluating emotions, especially negative ones^48^. The study of Prkachin et al.^48^ related to the relation between alexithymia and the perception of emotions shows that subjects with high levels of alexithymia are not able to evaluate the intensity of emotions in particular in relation to fear.

A possible explanation is related to the differences in coding and judging processes^56,57^. While the first one is an automatic process able to detect stimuli, the second is related to a more cognitive evaluation. It means that alexithymic subjects are able to detect emotions but they are not able to evaluate them. In this line, our results show that alexithymic subjects tend to be anchored to basic emotions, attributing negative emotions (i.e. fear) to a high percentage of emotional situations.

In relation to negative emotions, the study of Scarpazza et al.^58^, using the Visual Remapping of Touch (eVRT) emotional paradigm show that alexithymia is associated with difficulties in mapping emotions into one’s sensorial system, especially fear. Moreover, Barchetta et al.^59^ recently showed that difficulties in identifying and describing feelings and emotions are associated with a negative bias for past and present events.

From a neural point of view, a recent study by Pouga et al.^60^ using fMRI in order to investigate the neural basis in the individual differences related to socio-affective skills, show a significant correlation between people with high levels of alexithymia and the activity of the cingulate cortex. In particular related to the rostral anterior (arACC) and rostral posterior (prACC) cortex. While the arACC is related to affective taks, the prACC is related to cognitive tasks^61,62^. Therefore, the relation between alexithymia, the arACC and prACC could suggest a poor efficiency in the interaction between affective and cognitive processes^61^.

### Bottom line

In conclusion, our study shows a poor efficiency of Ekman’s model in explaining emotional situations of daily life. Here subjects with low levels of alexithymia tend to use the label “other” much more often than specific emotions. Furthermore, there is no significant degree of agreement between subjects.

On the other hand, our results show that subjects with high levels of alexithymia tend to use emotional labels more often than subjects with low levels of alexithymia. This leads us to hypothesize that alexithymia (literally “lack of words for emotions”) leads subjects to remain more anchored to the six basic emotions.

Based on our results, which spot foundational issues in existing emotion models, and the need to integrate or extend them to represent realistic emotional situations, we are designing a new model for emotions, using artificial intelligence methods. We intend to enhance and integrate emotion models, datasets and lexical resources, in order to make computational sense of everyday emotions. Methods include representing emotion-oriented resources as knowledge graphs, and using neuro-symbolic systems that take advantage of the explainability of graphs, of automated reasoning performed on them, as well as of the learnability of emotional patterns out of multimodal resources. This method has produced a first integrated formal ontology of emotions, the Emotion Frame Ontology, which provides an abstraction over existing emotion models, and a first-order theory to jointly represent both real world situations and emotional states (< owl:imports rdf:resource = "https://w3id.org/framester/prep/prepont/"/ >).

### Limitations

Our sample has a pronunced gender imbalance in the participants (94 female and 20 male) which is a limitation in generalizing our results which will be addressed in future works.

Since tweets have limited means to express real-life situations and associated emotions, future work may include a more traditional corpus annotated with Ekman’s emotions, in order to verify whether the inter-rater agreement improves with respect to our finding on the tweets corpus.

Our data collection through online questionnaires can reduce the influence linked to the interaction with the interlocutor, but it could lead to some biases in the participants, such as self-selection bias or social desirability bias.

## Supplementary Information

Supplementary Tables.

## Data Availability

Data is available by contacting the corresponding author.
