# Supplementary material for: Experiments on real-life emotions challenge Ekman's model
Source: Sci Rep. 2023 Jun 12;13:9511. doi: 10.1038/s41598-023-36201-5 (PMC10261107; doi:10.1038/s41598-023-36201-5)
Supplement: Supplementary file 1 — Supplementary Tables. [file 41598_2023_36201_MOESM1_ESM.docx]

| **DOS Entry ID** | **Emotion Name** | **Original Definition** | **Italian translation** |
| --- | --- | --- | --- |
| DOS1 | onism | The awareness of how little of the world you'll experience | La consapevolezza di quanto poco del mondo farai esperienza |
| DOS2 | funkenwangsvors  tellung | the primal trance of watching a campfire in the dark | La trance primordiale che si prova guardando un falò nel buio |
| DOS3 | ballagàrraidh | the awareness that you are not at home in the wilderness | La sensazione di non sentirsi a casa nella natura selvaggia e incontaminata |
| DOS4 | covalent bond | a moment of sudden involvement in a stranger's personal life that shatters the invisible glass box that usually surrounds us in public | Un momento di improvviso coinvolgimento nella vita personale di uno sconosciuto che frantuma il vetro invisibile che solitamente ci circonda in pubblico |
| DOS5 | anechosis | a state of exhaustion with continually being told what you want to hear | Uno stato di sfinimento provocato dal sentirsi dire quello che si vuole sentire |
| DOS6 | hailbound | mysteriously compelled to wave to passing strangers on a country road or a mountain path | L’inspiegabile bisogno di salutare gli sconosciuti che incrociamo passeggiando in una strada di campagna o in un sentiero di montagna |
| DOS7 | sonder | the awareness that everyone has a story | La consapevolezza che ognuno ha una propria storia |
| DOS8 | thrapt | awed at the impact someone has had on your life, feeling intimidated by how profoundly they helped shape your identity | Il senso di disorientamento dovuto alla consapevolezza dell’impatto che alcune persone hanno avuto sulla nostra vita, e quindi sentirsi intimiditi da quanto profondamente ci hanno aiutato a dare forma alla nostra identità |
| DOS9 | attriage | the state of having lost all control over how you feel about someone | Perdere il controllo sui sentimenti che provi per una persona |
| DOS10 | drisson | an unexpected twinge of attraction for a friend, a flutter of desire you don't necessarily want to feel, that didn't even seem possible up to this point | L'inaspettata fitta di attrazione per un amico o un'amica, un fremito di desiderio che non vuoi necessariamente sentire, e che non ti è mai sembrato possibile sentire fino a questo punto |

**Supplementary Table S1:** Original and edited entries of DOS

.

| **ELTEA Entry ID** | **Associated emotion** | **Original content** | **Italian Translation** |
| --- | --- | --- | --- |
| ELTEA1 | Sad | All these negative emotions are nothing but distortions of love. And this is because of a lack of wisdom. | Realizzare che alcune emozioni negative non sono altro che distorsioni dell’amore. E il motivo di tale distorsione è una mancanza di saggezza. |
| ELTEA2 | Surprise | I hate reading books. I've never read a book. Never. But once you give me a book I'll read it because you give it to me. | Quando odi leggere e infatti non hai mai letto un libro in vita tua ma saresti disposto a leggerne uno se qualcuno te lo regalasse, per il semplice fatto che ti è stato regalato. |
| ELTEA3 | Happiness | Well good. It's been a very pleasant discussion and you've been a good sport. Hope to speak again soon. | La speranza di parlare di nuovo con qualcuno il prima possibile perché l’ultima volta che avete parlato è stato particolarmente piacevole. |
| ELTEA4 | Fear | I have a hard time expressing my feelings because 99% of the time I don't even know what I'm feeling. | La difficoltà nell'esprimere i propri sentimenti dovuta all'incapacità di comprenderli che caratterizza la maggior parte della propria esperienza vissuta. |
| ELTEA5 | Happiness | Everything has its beauty. But not everyone see it. | Ogni cosa possiede una bellezza propria, ma non tutti riescono a vederla. |
|  |  |  |  |
| ELTEA6 | Disgust | When you can say 'I god damn love you!' to everyone but your bf because he has a meltdown & now you're dead inside & hate his guts. | Quando puoi dire "ti amo da morire" a chiunque tranne la persona con cui hai una relazione romantica perché dopo averglielo detto ha un esaurimento nervoso e tu ti senti morire dentro odiandola profondamente. |
| ELTEA7 | Surprise | They all hate on you because you are different and they are jealous of you | Rendersi conto che l'odio degli altri nei tuoi confronti dovuto al fatto che appari diverso è in realtà dovuto all'invidia che provano nei tuoi confronti. |
| ELTEA8 | Anger | I never want to hurt anyone's feelings. That's my problem I'd feel better if I stopped letting people walk all over me & I cussed them out. | Il desiderio di non voler ferire i sentimenti degli altri ma con la consapevolezza che sia un problema in quanto staremmo meglio se smettessimo di accettare di essere calpestati da alcune persone. |
| ELTEA9 | Fear | How can someone make me so sad but still I only want them to stay I wanna say I love you so bad but I don't wanna scare you away. | L'impossibilità di spiegarsi il desiderio di tenersi vicino la persona amata nonostante ci faccia male, il desiderio di dirle "ti amo da morire ma non voglio spaventarti e allontanarti da me. |
| ELTEA  10 | Disgust | Some days I'm so needy and need a lot of reassurance and other days I just need space and get annoyed so quickly, so idk. | L'inspiegabile alternanza di giorni in cui hai bisogno di rassicurazioni e attenzioni e di giorni in cui desideri spazio e qualsiasi cosa ti infastidisce. |

**Supplementary Table S2**: Original and edited entries of ELTEA17
